# Sphingosine 1 phosphate receptor-1 (S1P1) promotes tumor-associated regulatory T cell expansion: leading to poor survival in bladder cancer

**DOI:** 10.1038/s41419-018-1298-y

**Published:** 2019-01-18

**Authors:** Yi-Na Liu, Han Zhang, Lin Zhang, Ting-Ting Cai, Dai-Jia Huang, Jia He, Huan-He Ni, Fang-Jian Zhou, Xiao-Shi Zhang, Jiang Li

**Affiliations:** 1 Collaborative Innovation Center for Cancer Medicine, State Key Laboratory of Oncology in South China, Sun Yat-sen University Cancer Center, Guangzhou, 510060 China; 2 Department of Biotherapy, Sun Yat-sen University Cancer Center, 651 Dongfeng East Road, Guangzhou, 510060 China; 3 Department of Urology, Sun Yat-sen University Cancer Center, 651 Dongfeng East Road, Guangzhou, 510060 China

## Abstract

Regulatory T cells (Tregs) represent an important contributor to cancer immune escape, but the molecular mechanism responsible for Treg expansion in tumors is heterogeneous and unclear. Here, we investigated the role of S1P1, a receptor of the bioactive lipid sphingosine 1-phosphate (S1P), in regulating the crosstalk between tumor cells and tumor-associated Tregs in bladder cancer (BC). We found that the frequency of CD4^+^Foxp3^+^ Tregs was increased in circulating and tumor-infiltrating lymphocytes from BC patients. S1P1 expression was upregulated in BC tissues compared with tumor-adjacent tissues and was positively correlated with the density of tumor-infiltrated Foxp3^+^ Tregs. Both S1P1 and Treg predicted poor overall survival in BC patients. The in vitro data paralleled the in vivo data and suggested that the activation or overexpression of S1P1 in BC cells promoted the generation of BC-induced (i)Tregs from CD4^+^CD25^−^cells, and the generation of these cells was reversed by treatment with anti-IL-10 or anti-TGF-β. Moreover, S1P1 promoted Treg migration mediated by BC cells. Mechanistically, S1P1 activated the TGF-β signaling pathway, leading to the secretion of TGF-β and IL-10 from BC cells. In total, our findings suggest that S1P1 induces tumor-derived Treg expansion in a cell-specific manner and serves as a potent prognostic biomarker and therapeutic target in BC.

## Introduction

Bladder carcinoma (BC) is the fifth most common cancer, accounting for 85–90% of primary carcinomas, and its incidence is increasing worldwide^1,2^. Notably, patients with BC show evidence of acquired immune dysfunction, particularly the expansion of regulatory T cells (Tregs)^3^. However, the tumor-infiltrated Tregs include heterogeneous subsets of cells expressing different immunosuppressive molecules favoring tumor progression, such as CTLA-4, PD-1, LAG-3, TIM-3, and TIGIT. The detailed molecular mechanism responsible for Treg expansion in cancers is heterogeneous and remains poorly understood.

Sphingosine 1-phosphate (S1P), a potent bioactive lipid, exerts many biological effects on different types of cells, including normal cells, and these effects include changes to cell migration, proliferation, and angiogenesis^4^. There are five types of G-protein-coupled S1P receptors, and among these receptors, S1P1-3 are the most widely expressed^5^. In addition, S1P can promote the motility, survival, growth, and transformation of cancer cells through multiple pathways^6^. It was recently reported that S1P signaling maintains the mitochondrial content of naive T cells to support their constant migration, and the expression of sphingosine 1-phosphate receptor 1 (S1P1, encoded by the S1PR1 gene) in T cells inhibits the generation of Tregs but reciprocally drives the development of type 1 T helper (Th1) cells^7^. However, in this study, we observed that extensive S1P1 expression in BC tissues was positively associated with the number of tumor-infiltrated Tregs, and the levels of both S1P1 and Treg showed prognostic implications in BC patients. Mechanistic analyses revealed that S1P1 promoted BC-associated (i)Treg induction and (n)Treg recruitment in vitro through tumor-derived TGF-β and IL-10 secretion. In summary, these findings uncover tumor-cell-specific S1P1 function, namely, the induction of tumor-associated Treg expansion in BC, and suggest that S1P1 serves as a potential prognostic biomarker and therapeutic target for BC patients.

## Results

### Increased S1P1 expression is associated with regulatory T cell expansion in BC

**Fig. 1:**
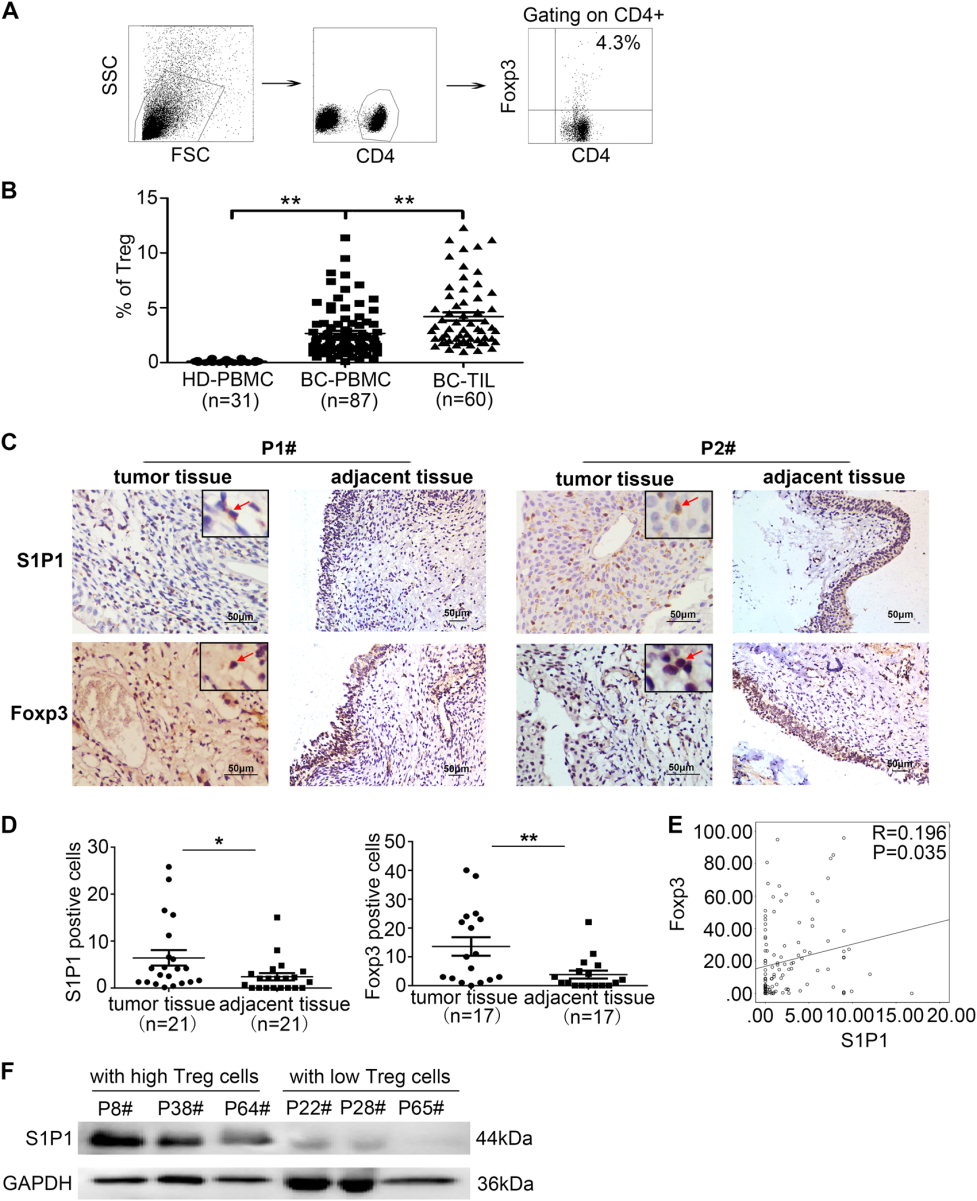
Correlation of S1P1 expression with numbers of Foxp3^+^ Tregs in tumor specimens from BC patients. **a** Gating strategy for the assessment of the Treg population by flow cytometry. The CD4^+^ cells were gated from live PBMCs or TILs, and the CD4^+^Foxp3^+^ cells were further gated as Tregs. **b** A statistical analysis revealed that the percentage of Treg cells in the population of TILs (n = 60) was significantly higher compared with that in the population of PBMCs (*n* = 87) from BC patients (*P* < 0.01), whereas the percentage of Tregs in the population of PBMCs (*n* = 87) from BC patients was significantly higher than that in the PBMCs from healthy donors (*P* < 0.01, *n* = 31). **c** Representative IHC staining for Foxp3 and S1P1 in tumor and tumor-adjacent tissues from BC patients. **d** Statistical analysis of the levels of tumor-infiltrated Foxp3^+^ cells and S1P1 in tumor tissues and tumor-adjacent tissues from the same patient (*P* < 0.05; n = 17 or 21, respectively). **e** Pearson’s correlation coefficient and linear regression array for the correlation of the number of tumor-infiltrated Foxp3^+^ cells and the tumor S1P1 expression level (*R* = 0.196, *P* = 0.035). **f** Immunoblotting analysis of the levels of S1P1 in tumor tissues obtained from BC patients with high numbers of tumor-infiltrating Foxp3^+^ cells and BC patients with lower numbers of tumor-infiltrating Foxp3^+^ cells. IHC, immunohistochemical staining; *R*, Spearman’s correlation; *P*, significance of the correlation

We observed that the frequency of CD4^+^Foxp3^+^ Tregs was significantly increased in the populations of circuiting and tumor-infiltrating T cells from BC patients compared with those from healthy donors, as demonstrated by flow cytometry (Fig. 1a, b, *P* < 0.05). Moreover, the S1P1 expression level and Foxp3^+^ Treg density were substantially increased in BC tissues compared with tumor-adjacent tissues (Fig. 1c, d, *P* < 0.05), and interestingly, tumor S1P1 expression was significantly correlated with the number of Foxp3^+^ Tregs in tumor specimens from 116 BC patients (Fig. 1e, *P* = 0.035, *R* = 0.196). We further demonstrated that the level of S1P1 was increased in tumor tissues from BC patients with a higher frequency of tumor-infiltrating CD4^+^Foxp3^+^ Tregs than in those from BC patients with a lower frequency of tumor-infiltrating CD4^+^Foxp3^+^ Tregs (n = 3, Fig. 1f). These data indicate that S1P1 is widely expressed in BC tissues and is positively associated with Treg density in BC patients and thus suggest that S1P1 plays a role in regulating Treg expansion in BC.

### S1P1 promotes functional BC-induced CD4^+^CD25^high^Foxp3^+^ (i)Treg generation in vitro

**Fig. 2:**
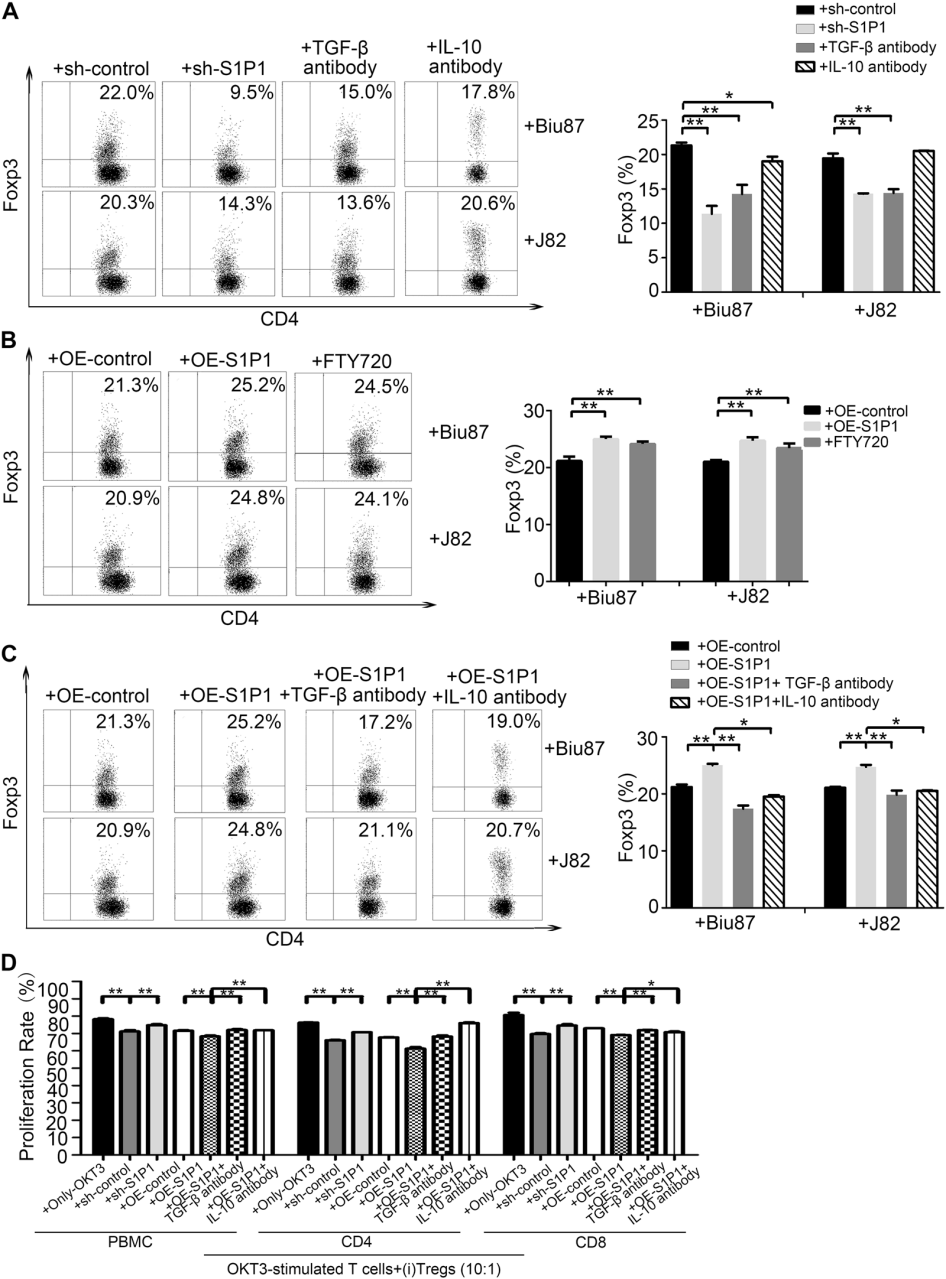
Promotion of the induction of tumor-associated (i)Tregs by S1P1 in vitro. **a**–**c** CD4^+^ cells and BC-derived cells were cocultured, after the BC-derived cells were treated with lenti-shS1P1-vector or with anti-TGF-β or anti-IL-10 antibodies (**a**), lenti-S1P1-expressing vector and S1P1 agonist (FTY720) (**b**), or lenti-S1P1-expressing vector and either anti-TGF-β or anti-IL-10 antibody (**c**) for 48 h. The percentage of CD4^+^Foxp3^+^Tregs were measured by FACS. **d** A statistical analysis showed the proliferation of OKT3-stimulated PBMCs, including CD4 and CD8 cells, after coculture with (i)Treg cells induced from BC-derived cells that were treated with lenti-shS1P1-vector, lenti-S1P1-expressing vector, lenti-control vector or lenti-S1P1-expressing vector combined with either TGF-β or IL-10 antibody for 5 days. The data shown were obtained from one of three experiments, and the statistical analyses were performed using Student’s *t*-test. The bars represent the SEMs from three experiments. *P < 0.05. **P < 0.01

It has been proposed that the accumulation of Tregs in tumor microenvironments is caused by increases in the recruitment of natural (n)Tregs from peripheral blood and the tumor-cell-mediated induction of (i)Tregs from CD4^+^CD25^−^T cells^8^. We found that the BC-derived cell lines J82 and Biu87 could induce CD4^+^CD25^+^Foxp3^+^ (i)Tregs from CD4^+^CD25^−^T cells in a coculture system (Fig. S1A and B). To further investigate the role of S1P signaling in BC-derived induced (i)Treg generation, we first generated BC-derived cells (J82 and Biu87) that ectopically expression of S1P1 through lenti-S1P1-expressing vector or lenti-shS1P1-vector transfection. Compared with the percentage of (i)Tregs induced by BC-derived cells following the administration of shControl, the percentage of CD4^+^Foxp3^+^ (i)Tregs induced by BC-derived cells was significantly reduced (*P* < 0.05) following the administration of siRNA-S1P1 or shRNA-S1P1 or in the presence of anti-TGF-β and anti-IL-10 antibodies and was inversely increased by forced S1P1 expression or in the presence of FTY720 (Fig. 2a, b, and Fig. S1c and d). Interestingly, the presence of anti-TGF-β or anti-IL-10 antibody reversed the promotion of (i)Tregs induced by BC-derived cells with S1P1 overexpression (Fig. 2c). The suppressive function of (i)Tregs induced by BC-derived cells with S1P1 overexpression was sharply stronger than that of (i)Tregs induced by shControl-treated BC-derived cells, whereas the suppressive function of (i)Tregs induced by BC-derived cells following the administration of shS1P1 or TGF-β antibody was weaker than that of (i)Tregs induced by shControl-treated BC-derived cells (Fig. 2d and fig. S1c). In total, these results suggest that S1P1 promotes BC-driven (i)Treg generation through the effect of the cytokines TGF-β and IL-10.

### S1P1 promotes BC-derived CD4^+^Foxp3^+^ Treg migration in vitro

**Fig. 3:**
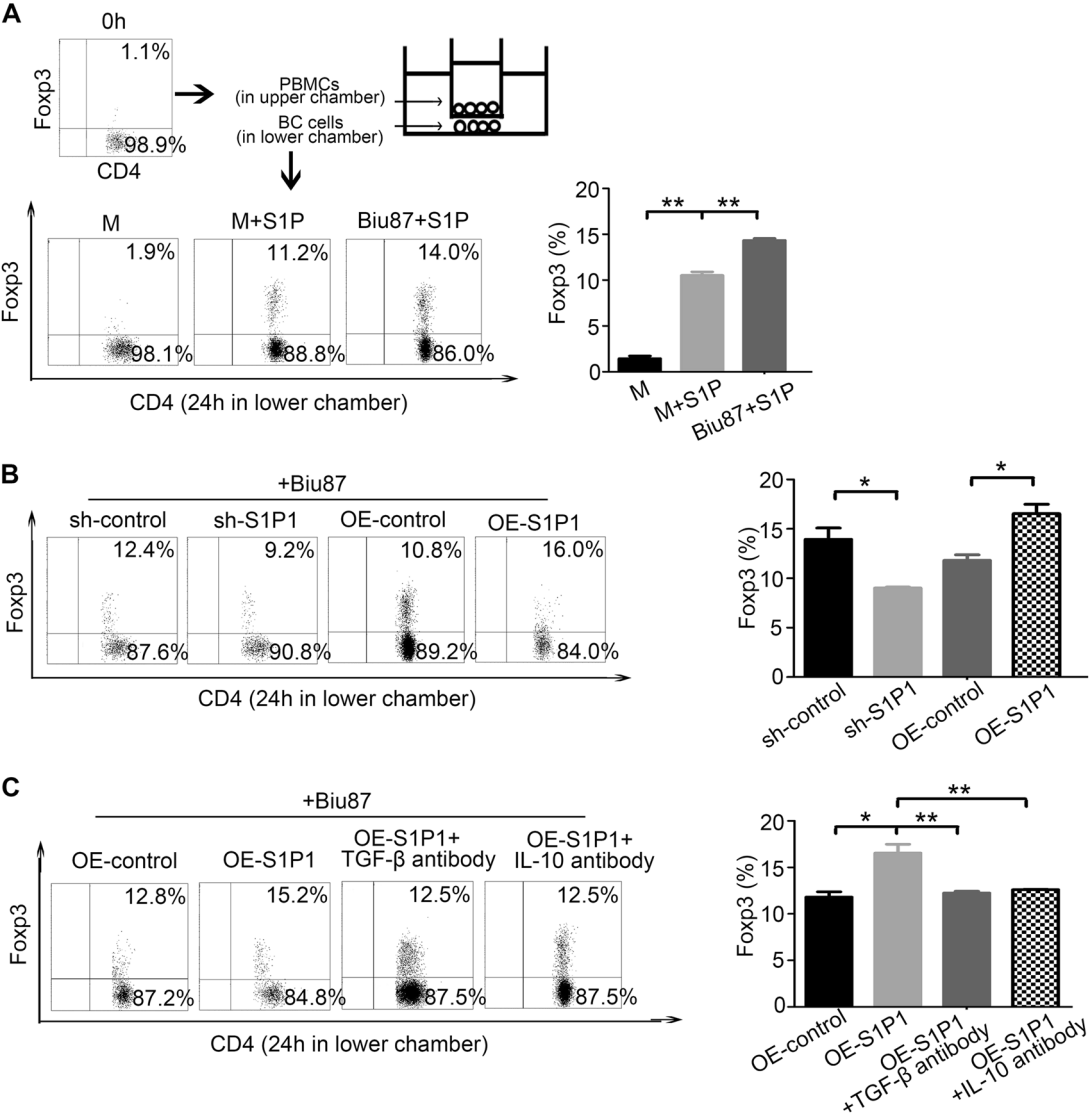
S1P1 increases the migration of (n)Tregs in BC microenvironments. **a**–**c** Migration of CD4^+^Foxp3^+^ cells in PBMCs after coculture with Biu87 and S1P or in medium with S1P (**a**), Biu87 cells transfected with lenti-shS1P1 vector, lenti-S1P1-expressing vector or the corresponding lenti-control vector (**b**), or Biu87 cells transfected with lenti-S1P1-expressing vector, lenti-S1P1-expressing vector and TGF-β antibody or with lenti-S1P1-expressing vector and IL-10 antibody (**c**) in a Transwell system for 24 h. Representative FACS staining data are shown. The statistical analysis were performed using the Student’s *t*-test. The bars represent the SEMs from three experiments. *P < 0.05, **P < 0.01

The expansion of Tregs in tumor microenvironments is partially caused by an increased recruitment of (n)Tregs from the peripheral blood, and we found that the serum level of chemokines, including CCL19, IL-8, CXCL1 and CXCL12 (binding to CXCR2), and RANTES (binding to CCR5), was significantly increased in BC patients (*P* < 0.001, Fig. S2A). Among these chemokines, RANTEs was implicated in the mediation of Treg migration in the previous studies^9,10^. Considering that the S1P signaling generally promotes the exit of lymphocytes from the spleen or lymph node to the blood^11,12^, we further investigated whether S1P/S1P1 signaling could promote the recruitment of natural (n)Tregs into BC tissues. Using a Transwell System, we found that human recombinant S1P protein promoted not only the migration of Tregs but also the migration of Tregs mediated by BC-derived cells in vitro (Fig. 3a). However, the knockdown of S1P1 decreased the migration of Tregs mediated by BC-derived cells, whereas the ectopic overexpression of S1P1 promoted the migration of Tregs mediated by BC-derived cells (Fig. 3b). Interestingly, the promotion of Treg migration induced by J82-S1P1 was reversed in the presence of anti-TGF-β or anti-IL-10 antibody (Fig. 3c). In addition, we determined that the level of S1P1 was increased in BC-derived cell lines, including EJ, T24, Biu87, and J82, after treatment with S1P (fig. S3B). In total, these observations suggest that an increased recruitment of (n)Treg to tumor microenvironments in BC for the activation of S1P signaling and increased chemokine levels such as RANTEs.

### S1P1-mediated (i)Treg cell generation is correlated with activation of the TGF-β signaling pathway

**Fig. 4:**
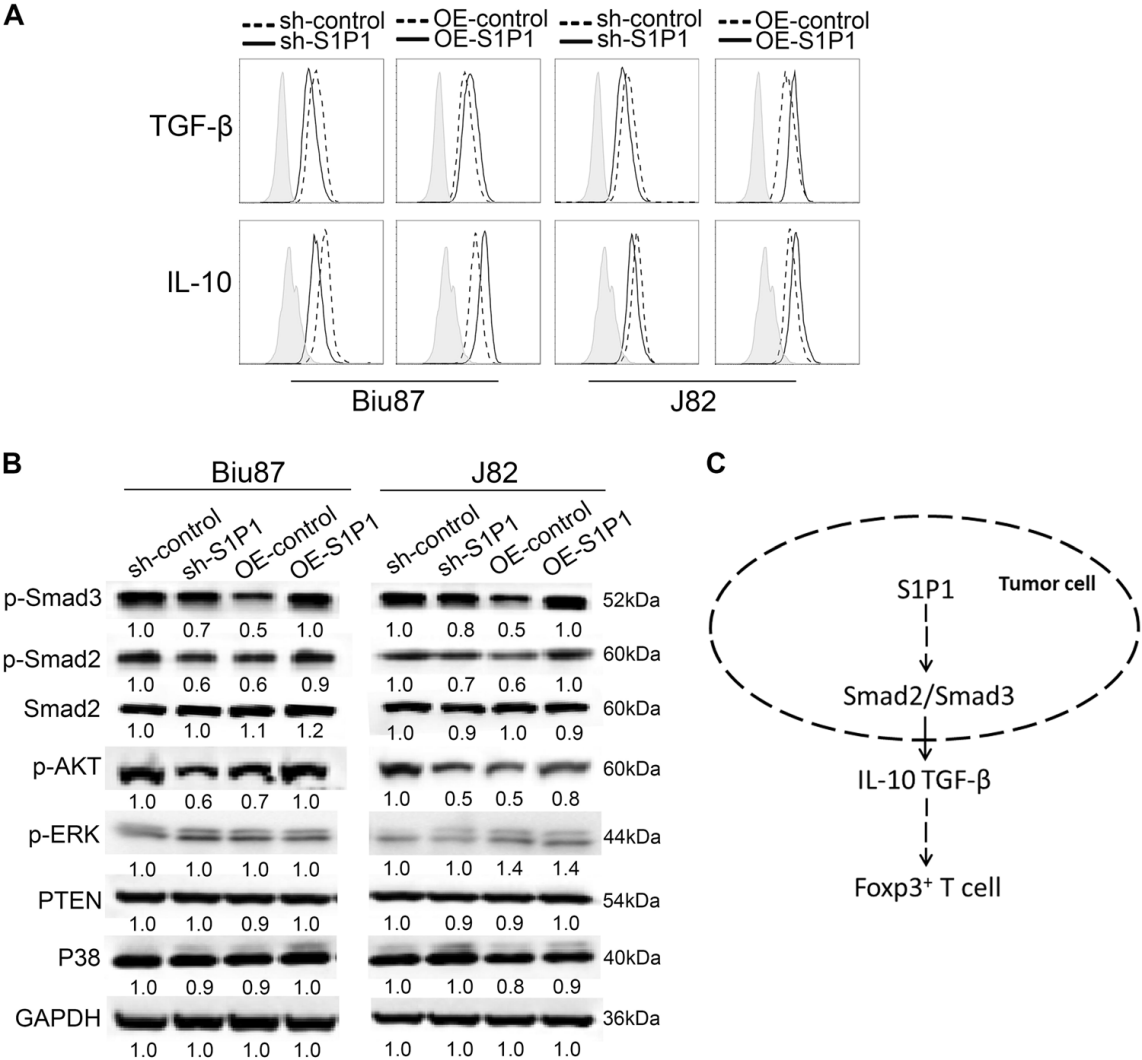
S1P1-mediated BC-induced (i)Treg generation is associated with activation of the TGF-β/Smad2/3 signal pathway. **a** The production of IL-10 and TGF-β by BC-derived Biu87 and J82 cells following treatment with shS1P1, lenti-S1P1-expressing vector and the corresponding control vectors was measured using a flow cytometer, and representative data from one of three independent experiments are shown. **b** Immunoblotting results for the levels of p-Smad3, p-Smad2, Smad2, p-AKT, p-ERK, PTEN, and P38 in the Biu87 and J82 cells after treatment with lenti-shS1P1 vector, lenti-S1P1-expressing vector and the corresponding lenti-control vectors; GAPDH was included as a control. **c** Working model of the S1P signaling-mediated regulation of the expansion of tumor-associated Tregs

We observed that the levels of TGF-β and IL-10 were increased in BC-derived cells with S1P1 overexpression but decreased in BC-derived cells with S1P1 depletion compared with the corresponding control cells (Fig. 4a). Mechanistic studies revealed that the overexpression and depletion of S1P1 in BC-derived cells increased and decreased the levels of both p-Smad2 and p-Smad3, respectively, and the levels of p-AKT, PTEN, P38, and p-ERK were not altered in BC-derived cells with ectopic S1P1 expression (Fig. 4b). These data suggest that S1P1 signaling induces the production of TGF-β and IL-10 and that this induction is associated with the activation of TGF-β signaling in BC-derived cells and leads to the expansion of Tregs in tumor microenvironments (Fig. 4c).

### The levels of S1P1 and tumor-infiltrated Foxp3^+^ Treg have prognostic implications in BC

We summarize the correlations of the Foxp3^+^ Treg density or S1P1 expression with the clinicopathological parameters of BC patients in Table 1. Briefly, BC patients at an advanced T stage showed a notably higher number of Foxp3^+^ Tregs and an increased S1P1 level in the tumor microenvironment (*P* = 0.002 and 0.008, Table 1), whereas no significant correlations were observed between the density of Foxp3^+^ Tregs or S1P1 expression and the other clinical parameters, including N stage, M stage, grade stage or treatment model (*P* > 0.05).

**Table 1:** Association of the frequency of Foxp3^+^ cells and S1P1 expression in tumor tissues with the clinical parameters of 116 patients with bladder carcinoma in 2003–2004

Clinicopathological parameter	High level of Foxp3^+^ (%)	*P-*value	High level of S1P1^+^ (%)	*P*-value
*Age*				
<60 y	34 (54.0)	0.445	32 (50.8)	0.568
≥60 y	27 (50.9)		27 (50.9)	
*Gender*				
Female	50 (53.8)	0.390	47(50.5)	0.537
Male	11 (47.8)		12 (52.2)	
*T status*				
NMI	23 (39.0)	0.002*	23 (39.0)	0.008*
MI	38 (66.7)		36 (63.2)	
*N status*				
0	56 (53.9)	0.310	51 (49.0)	0.198
1–3	5 (41.7)		8 (66.7)	
*M status*				
0	57 (53.3)	0.434	55 (51.4)	0.478
1–2	4 (44.4)		4 (44.4)	
*Grade*				
G1	24 (51.1)	0.467	18 (38.3)	0.020*
G2–G3	37 (53.6)		41 (59.4)	
*Multiplicity*				
Unifocal	59 (54.6)	0.105	56 (51.9)	0.339
Multifocal	2 (25.0)		3 (37.5)	
*Treatment model*				
TURBT	39 (53.4)	0.482	35 (47.9)	0.266
RC	22 (51.2)		24 (55.8)	

*NMI* nonmuscle invasive bladder cancer, *MI* muscle invasive bladder cancer, *TURBT* transurethral resection of bladder tumor, *RC* radical resection of bladder cancer; *means *P* < 0.05

**Fig. 5:**
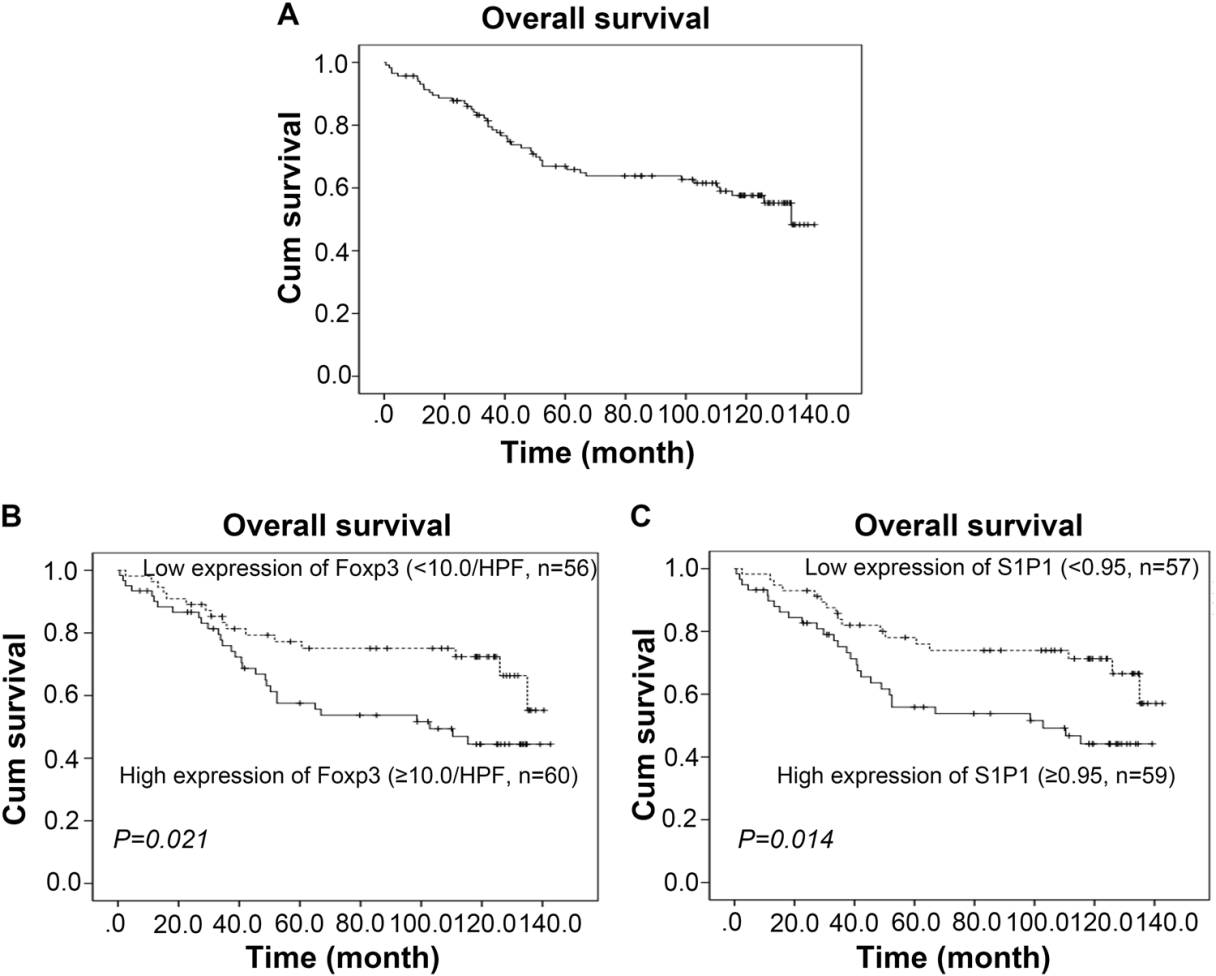
Prognostic values of tumor-infiltrating Foxp3^+^ cells and tumor S1P1 expression in BC patients. **a** Overall survival (OS) curve of 116 BC patients. **b**–**c** The levels of tumor-infiltrating Foxp3^+^ cells (**b**) and S1P1 (**c**) were significantly negatively associated with the OS of BC patients (*P* = 0.021 and 0.014, respectively, log-rank test), as demonstrated by the Kaplan–Meier survival curves of OS. The cutoff value is the median Foxp3^+^ cell density or S1P1 expression

The median survival time of the 116 patients with BC was 87 months (range 0–132 months), and 46 patients had died by the time of the last follow-up. The cumulative OS rates at the 5- and 10-year follow-up of the patients included in the present study were 77.7% and 65.5%, respectively (Fig. 5a). Moreover, a high Foxp3^+^ Treg number or S1P1 level was significantly associated with a reduced OS (*P* < 0.05, Fig. 5b, c), as demonstrated through Kaplan–Meier and log-rank test analyses. However, as expected (and as shown in Table S1), clinicopathological parameters such as age (HR, 3.815; 95% CI, 2.043–7.124; *P* < 0.001), tumor (T) status (HR, 4.456; 95% CI, 2.255–8.802; *P* < 0.001), clinical grade (HR, 3.318; 95% CI, 1.642–6.701; *P* < 0.001) and treatment model (HR, 2.220; 95% CI, 1.243–3.965; *P* = 0.001) also has prognostic value. A multivariate Cox model analysis found that exception of the classical prognostic factors, such as age (HR, 3.733; 95% CI, 1.957–7.275; *P* < 0.001) and T status (HR, 3.459; 95% CI, 1.400–8.547; *P* = 0.007), the levels of Foxp3+ Tregs or S1P1 expression were not independent predictors of OS (Table S1, *P* > 0.05). In summary, these data indicate that the levels of both S1P1 and Tregs in tumor microenvironments might have prognostic value for the malignant progression of BC.

## Discussion

In normal tissues, the net effect of S1P signaling via the S1PRs is the regulation of a wide range of cellular processes, such as cell survival, motility, and angiogenesis, and deregulation of the S1P signaling pathway contributes to the development and progression of cancers. There is strong evidence linking the involvement of S1P receptors (S1PRs) in cancer progression, and the oncogenic effects of S1P can result from alterations in the expression of one or more of the S1PRs and/or the enzymes that regulate the levels of S1P. These studies have led to clinical trials in patients with various types of tumors evaluating the potential of therapeutic strategies targeting S1PRs. For example, FTY720 was approved by the US-FDA in 2010 for the treatment of multiple sclerosis under the trade name Gilenya, and there is increasing interest in the potential repurposing of fingolimod for cancer treatment. However, the role of S1PRs and related signaling pathways in the development of cancers and cancer-associated chronic inflammation remains elusive. The analysis of the clinical samples included in this study indicated that S1P1, one of the receptors that initiate S1P signaling, is widely expressed in BC tissues and positively associated with the density of tumor-infiltrated Tregs. Importantly, both S1P1 and Treg exhibit prognostic value for BC patients.

Identification of the molecular mechanism underlying Treg accumulation in tumor microenvironments will help to overcome Treg-induced immunosuppression and boost effector T-cell responses. Previous studies have shown that the Treg population is often expanded in cancer host, and Treg frequencies are often higher in tumor tissues than in the blood or lymphoid organs^13–16^. This study revealed that the CD4^+^Foxp3^+^ Treg frequency is increased in both the peripheral blood and tumor biopsies from BC patients compared with healthy controls and is highest in BC tissues. Over the past decade, increasing evidence has suggested that the expansion of Tregs in tumor biopsies primarily reflects the (i)Tregs from CD4^+^CD25^-^ naïve T cells educated by tumor cells^17,18^. Many molecules and signaling pathways, including TGF-β and IL-10^19–22^, have been associated with the generation of (i)Tregs in cancer. Here, we found that S1P1 promotes BC cell-induced (i)Treg generation from CD4^+^CD25^−^ cells and that this increase could be inhibited by anti-IL-10 and anti-TGF-β antibodies. However, recent studies showed that the intrinsic expression of S1P1 in T cells inhibits Treg differentiation while driving Th1 differentiation by attenuating the function of TGF-β−Smad3 via mTOR signaling^23,24^, and high S1P1 expression has been associated with various autoimmune diseases^25,26^. However, some researchers have reported that impaired S1P1 phosphorylation enhances TH17 polarization^27–29^. Consistent with these reports, we found that the depletion of S1P1 in OKT3-stimulated T cells promoted the differentiation of CD4^+^Foxp3^+^ Tregs but decreased the differentiation of CD4^+^IFNγ^+^ Th1 and CD4^+^IL-17^+^ Th17 cells, whereas forced S1P1 expression in OKT3-stimulated T cells exerted the inverse effect on the differentiation of Treg, Th1 and Th17 cells (Fig. S3). These results differ from the effect of tumor S1P1 expression on tumor-associated (i)Treg induction observed in our study. Thus, we deduce that S1P1 regulates the induction of tumor-associated Tregs in a cell-specific manner in BC.

S1P1 functions in the egress of T cells from the lymph node^30^. Specifically, it has been reported that S1P1 directs the exit of T cells from the spleen into the blood and from lymph nodes and Peyer’s patches into the lymph^31^. Here, we found that S1P and S1P1 facilitated the BC-mediated migration of Tregs in vitro, but the presence of anti-IL-10 and anti-TGF-β antibodies reversed the increase in the migration of Tregs from BC-derived cells with S1P1 overexpression. Previous studies have shown that S1P1 mediates the selective mobilization of tumor-specific Tregs from the bone marrow of breast cancer patients and their translocation into tumor tissue^27,32^. In contrast, the increased secretion of chemokines from tumor cells, such as RANTEs^33,34^, promotes the recruitment of (n)Tregs from circulating lymphocytes to tumor microenvironments. Here, we observed that the serum levels of various chemokines, including CCL19, IL-8, CXCL1, CXCL12, and RANTEs, the latter of which has been associated with the recruitment of Tregs in cancer patients^10,35^, were increased in BC patients.

A further mechanistic study revealed that S1P1 promotes the phosphorylation of Smad2/3 to activate the TGF-β signaling pathway and the production of IL-10 and TGF-β from BC cells. Indeed, S1P1 enhances the tumor-associated induction of (i)Tregs from naïve T cells by increasing the production of TGF-β and IL-10 in the tumor environment. Based on these data, we hypothesize that the activation of TGF-β signaling mediated by S1P1 is associated with Treg expansion in BC.

S1P1 expression in tumor specimens exhibits a significant positive association with the tumor-infiltrated Foxp3^+^ Treg density, and both of these features are associated with poor OS in BC patients. Our data suggest that S1P1 promotes tumor-associated (i)Treg expansion in a cell type-specific manner and that this effect is associated with the activation of TGF-β signaling and the production of TGF-β and IL-10 in BC cells (Fig. 4c). This knowledge might enable the prognostication of the BC disease course and provide potential targets of immunotherapy for BC patients.

## Materials and methods

### Patients and cell lines

Peripheral blood and/or fresh tumor tissues were collected from 97 patients who were newly diagnosed with BC at Sun Yat-sen University Cancer Center, Guangzhou, China, from February 2013 to March 2014. These patients did not receive any preoperative chemoradiotherapy. Thirty-one age-matched healthy donors were recruited to serve as controls. In addition, paraffin-embedded tumor tissues were collected from 116 patients who were newly diagnosed with BC at Sun Yat-sen University Cancer Center, Guangzhou, China, from 2003 to 2004. The clinical details of the patients are provided in Table 1. All the patients and healthy donors provided written informed consent prior to the collection of blood samples and/or the harvesting of tumors. The present study was approved by the Research Ethics Committee of the Sun Yat-sen University Cancer Center.

Human BC-derived cell lines, including EJ, T24, Biu87, and J82, were maintained under standard conditions, and all the cell lines were cultured in complete RPMI 1640 medium (Gibco, Shanghai, China) containing 10% fetal bovine serum (FBS, Excell Bio, S. America). The J82 cell line was purchased from ATCC (Shanghai, China) and was cultured in RPMI 1640 medium (Gibco) supplemented with 10% FBS (Excell Bio).

### Flow cytometry

Human monoclonal Abs against CD4, CD8, Foxp3, CD25, TGF-β, and IL-10 conjugated with different fluorescent dyes were purchased from BD Bioscience (San Jose, CA, USA) or eBioscience (San Diego, CA, USA). In the present study, Tregs from peripheral blood mononuclear cells (PBMCs) or tumor tissues were classified as CD4^+^Foxp3^+^ cells via fluorescence-activated cell sorting (FACS) using a multiplex gating strategy. Transcription factor staining was performed using a human regulatory T cell staining kit (eBioscience) and Foxp3 APC Abs (eBioscience). In brief, the harvested cells were washed and stained with surface phenotypic markers for 20 min on ice. After permeabilization and fixation, the cells were intracellularly stained with Fxop3 ABC Abs, and the positively stained cells were detected using a Beckman Coulter Gallios Flow Cytometer and analyzed using FlowJo V10 software.

### Immunohistochemistry

Paraffin-embedded tissues were continuously sectioned at a thickness of 4 µm. Immunohistochemical (IHC) staining for Foxp3 and S1P1 was performed using a primary monoclonal mouse anti-human Foxp3 Abs (Santa Cruz Biotechnology, CA, USA) or a rabbit anti-human S1P1 Abs (Abcam, Cambridge, UK) according to the manufacturers’ instructions. Two pathologists independently scored the slides based on the Foxp3^+^ cell number and S1P1 level in the tumor specimens. Specifically, the specimens were given a IHC score of 0, 1, 2, or 3 if < 5%, ≥ 5% and < 10%, ≥ 10% and < 50%, or ≥ 50% of the cells were positive for S1P1, respectively, and the Foxp3 staining data were obtained by counting the positively stained lymphocytes in 10 separate 400x fields. Mouse anti-human IgG1 (DAKO, Copenhagen, Denmark) or normal rabbit anti-human IgG (Abcam, Cambridge, UK) was used as a negative control.

### siRNA and lenti-shRNA vector transfection

To alter the S1P1 levels in J82 and BIU87 cells, the cell lines were cultured to 50–60% confluence and subsequently transfected with S1PR1-siRNAs. Specifically, chemically synthesized 19-nt siRNA duplexes were obtained from RiboBio Company (Guangzhou, China) and were transiently transfected into the bladder cells using Lipofectamine 2000 (Invitrogen, Carlsbad, CA, USA) according to the manufacturer’s instructions; a siRNA-targeting control gene (siNC) was included in the present study. The mRNA levels in the cells 24 or 48 h after transfection were measured using RT-PCR, and specific protein expression was assessed by Western blotting (WB). The following siS1PR1 primer sequences were used: siS1PR1_001 5′-CGCCTCTTCCTGCTAATCA-3′; siS1PR1_002 5′-CGGTCTCTGACTACGTCAA-3′; and siS1PR1_003 5′-CGCTGCTCAAGACCGTAAT-3′.

Scrambled control short hairpin RNA (shRNA, shControl) or S1P1-specific shRNA (shS1P1) were cloned into a lentivector (GeneCopoeia, Guangzhou, China). To generate recombinant lentivirus, the lentiviral expression construct and the packaging plasmid mix were cotransfected into 293T cells according to the manufacturer’s instructions. Vectors expressing either S1P1 short hairpin RNAs (shRNAs) or a scrambled shRNA were generated using the Sigma shRNA system according to the manufacturer’s instructions. The following human S1P1 shRNA primers were used: 5′-CCCGGACGAATTCTTCGAAATGGGGCCCACCAGCGTCCCGCTG-3′ (forward) and 5′-TGCGGATCACTAGTGCTAGCCTAGGAAGAAGAGTTGACGTTTCC-3′ (reverse).

### In vitro generation and analysis of the suppressive function of BC-induced (i)Tregs

CD4^+^ T-cells were isolated from PBMCs of healthy donors through negative sorting using microbeads (Miltenyi Biotec Company, Bergisch Gladbach, North Rhine-Westphalia, Germany), according to the manufacturer’s instructions. Subsequently, the CD4^+^ T cells were cocultured with irradiated BC-derived cells with S1P1 overexpression or depletion in OKT3 (R&D, Minneapolis, MN, USA)-precoated 48-well plates for 5 days to induce the generation of (i)Tregs in the presence or absence of anti-IL-10, anti-TGF-β1 (5 ng/ml, R&D, Minneapolis, MN, USA) and FTY720 (0.1 μM, Sigma-Aldrich, St. Louis, MO, USA). The percentages of Treg cells were determined using FACS analysis. For the suppressive analysis, PBMCs from healthy donors were labeled with carboxyfluorescein diacetate succinimidyl ester (CFSE, 10 μM, eBioscience), and the CFSE-labeled PBMCs were plated onto OKT3-coated 96-well plates and cocultured with different (i)Tregs at a ratio of 10:1 or in medium alone for 5 days. The PBMCs were subsequently harvested and stained for CD4, CD25, and Foxp3, and staining data were acquired and detected through FACS analysis.

### Chemotaxis assay

Chemotaxis assays were performed using 24-well plates with 5-μm-pore-size inserts (Costar/Corning, Corning, New York, NY, USA) according to the manufacturer’s instructions. A total of 1 × 10^6^ PBMCs in serum-free medium were loaded into the upper chamber, and 5 × 10^6^ BC-derived cells with S1P1 overexpression or depletion were plated onto the lower chamber alone or in the presence of human recombinant S1P (10 μM, Sigma-Aldrich, St. Louis, MO, USA) or IL-10 or TGF-β antibodies. After 24 h of incubation, the suspended cells were harvested and stained for Treg markers, including CD4 and Foxp3, and staining data were acquired and measured via FACS.

### Quantitative reverse-transcription polymerase chain reaction (qRT-PCR) and immunoblotting analysis

Total RNA from cells was extracted using the TRIzol reagent (Invitrogen, Carlsbad, CA, USA) according to the manufacturer’s instructions. RT-PCR was performed using the RevertAid First-Strand cDNA Synthesis kit (Thermo Scientific, Carlsbad, CA, USA) and Premix Taq^TM^ (TaKaRa Taq^TM^ Version 2.0 plus dye). The primer sequences are shown in Supplementary Table S3. All the experiments were repeated at least five times, and GAPDH mRNA expression was used as a control.

Immunoblotting was performed using standard methods, as previously described^36^. Anti-rabbit primary antibodies directed against p-ERK (pT202/Y204), p-AKT (pT308), p-Smad2, Smad-2, p-Smad3, PTEN, and P38 were purchased from Cell Signaling Technology (Trask Lane Danvers, MA, USA). Anti-rabbit antibodies directed against S1P1 were obtained from Abcam (Cambridge, UK). GAPDH (ProteinTech, Wu Han, Hu Bei, China) was used as a loading control.

### Multiplex cytokine production (ELISA)

The levels of various chemokines, including MIP-1α, MIP-1β, CCL19, IL-8, CXCL1, CXCL5, CXCL12, and RANTES, in serum samples from BC patients or healthy donors were measured using multiplex cytokine ELISA kits (Bio-Rad, Hercules, CA, USA) according to the manufacturer’s instructions.

### Statistical analysis

All in vitro experiments were performed in triplicate and were repeated at least three times. The numerical data are presented as the means ± standard errors of the mean (SEMs). Pearson’s chi-squared test was used to analyze the correlation between IHC variants, the frequency of circulating or tumor-infiltrating Tregs and the clinicopathological parameters of the patients. Representative experiments are shown in the figures. All the data analyses were performed using SPSS 19.0 (SPSS, Chicago, IL, USA) and GraphPad Prism 6 (La Jolla, CA, USA). The Kaplan–Meier and log-rank tests were used for the survival analyses, and the univariate and multivariate analyses were based on the Cox proportional hazards regression model. All cutoff values were obtained using X-tile (Version 3.6.1, Yale University, New Haven, CT, USA). *P* < 0.05 was considered to indicate statistical significance in the present study.

### Authenticity of the data

The authenticity of this article has been validated by uploading the key raw data to the Research Data Deposit (RDD) public platform (www. researchdata.org.cn), and these data are associated with the RDD approval number RDDB2018000390.

## Supplementary information

Figure legends and Tables

Figure S1 Generation and function of tumor-associated (i)Tregs in BC

Figure S2 Factors contributing to Treg recruitment in BC

Figure S3 Forced expression or depletion of S1P1 in T cells alters the differentiation of OKT3-stimulated T cells into Th1, Th17 and Treg cells
