# Supplementary material for: Sphingosine 1 phosphate receptor-1 (S1P1) promotes tumor-associated regulatory T cell expansion: leading to poor survival in bladder cancer
Source: Cell Death Dis. 2019 Jan 18;10(2):50. doi: 10.1038/s41419-018-1298-y (PMC6362099; doi:10.1038/s41419-018-1298-y)
Supplement: Supplementary file 1 — Figure legends and Tables [file 41419_2018_1298_MOESM1_ESM.docx]

**SUPPLEMENTARY MATERIAL**

**Figure S1. Generation and function of tumor-associated (i)Tregs in BC.** **A and B** CD4^+^ cells cocultured with BC-derived cells, including EJ, T24, Biu87 and J82 cells, for 48 h. In the representative data (**A**) and statistical analysis (**B**), the percentage of CD4^+^Foxp3^+^ cells was measured by flow cytometry, and CD4^+^ cells in medium were included as a control. **C** Representative FACS histogram indicating the proliferation of PBMCs, including CD4^+^ and CD8^+^ T cells, in a coculture with tumor-induced (i)Tregs at a ratio of 10:1 under different conditions for 5 days. (i)Tregs were induced by Biu87 cells in a coculture system after the administration of sh-S1P1, sh-control, OE-S1P1, OE-control and OE-S1P1 + TGF-β antibody for 48 h. **D-E** CD4^+^ cells and BC-derived cells treated with siRNA-S1P1 or si-control vector were cocultured for 48 h. As indicated in the representative data (**D**) and statistical analysis (**E**), the percentage of Tregs induced by J82 and Biu87 cells treated with si-S1P1 was significantly decreased. The bars represent the SEMs from three experiments. * *P* < 0.05, ** *P* < 0.01.

**Figure S2. Factors contributing to Treg recruitment in BC. A.** The serum levels of chemokines, including MIP-1α, MIP-1β, CCL19, IL-8, CXCL1, CXCL5, CXCL12 and RANTES, in BC patients (n =59) and healthy donors (n = 20), were measured via a multiplex ELISA array. **B** Immunoblotting analysis for S1P1 expression in BC-derived cell lines, including T24, EJ, J82 and Biu87 cells, with or without S1P treatment. C. The mRNA levels of TGF-b and IL-10 in BC-S1P1 cells and BC-control cells were measured by real-time RT-qPCR. The bars represent the SEMs from three experiments. * *P* < 0.05, ** *P* < 0.01.

**Figure S3.** **Forced expression or depletion of S1P1 in T cells alters the differentiation of OKT3-stimulated T cells into Th1, Th17 and Treg cells**. **A** The percentages of CD4^+^Foxp3^+^, CD4^+^IFN-γ^+^ and CD4^+^IL-17^+^ cells in a population of CD4+ cells treated with lenti-S1P1, lenti-shS1P1 or lenti-corresponding control vectors for 72 h were measured by flow cytometry after CD4^+^ T cells. Representative data are shown. **B** Statistical analysis of the percentages of CD4^+^Foxp3^+^, CD4^+^IFN-g^+^ and CD4^+^IL-17^+^ cells in a population of CD4^+^ cells treated with lenti-S1P1, lenti-shS1P1 or lenti-corresponding control vectors for 72 h. The bars represent the SEMs from three experiments. * *P* < 0.05, ** *P* < 0.01.

**Table S1. Univariate and multivariate Cox regression analyses for OS of 116 patients with bladder carcinoma**

| **Variables** |  | **Univariate analysis** | | |  | **Multivariate analysis** | |
| --- | --- | --- | --- | --- | --- | --- | --- |
|  |  | **HR (95% CI)** | ***P*-value** | |  | **HR (95% CI)** | ***P*-value** |
|  |  | **Overall survival** | | | | |  |
| Age (**<** 60/**≥** 60) |  | 3.815 (2.043-7.124) | | **0.000*** |  | 3.733 (1.957-7.275) | **0.000*** |
| Gender (Female/Male) |  | 0.878 (0.409-1.887) | | 0.739 |  |  |  |
| T status (NMI/MI) |  | 4.456 (2.255-8.802) | | **0.000*** |  | 3.459 (1.400-8.547) | **0.007*** |
| N status (0/1-3) |  | 1.297 (0.511-3.294) | | 0.585 |  |  |  |
| M status (0/1-3) |  | 1.885 (0.743-4.785) | | 0.182 |  |  |  |
| Grade (G1/G2-G3) |  | 3.318 (1.642-6.701) | | **0.001*** |  | 2.119 (0.890-5.047) | 0.090 |
| Multiplicity (Unifocal/Multifocal) |  | 0.916 (0.284-2.955) | | 0.883 |  |  |  |
| Treatment Model (TURBT/RC) |  | 2.220 (1.243-3.965) | | **0.007*** |  | 0.602 (0.281-1.292) | 0.193 |
| Expression of Foxp3 (Low/High) |  | 2.017 (1.096-3.710) | | **0.024*** |  | 1.747(0.849-3.593) | 0.130 |
| Expression of S1P1 (Low/High) |  | 2.116 (1.156-3.876) | | **0.016*** |  | 1.089 (0.512-2.315) | 0.826 |

**Notes:** *Significant difference. a, The expression of Foxp3 (Low/High) was based on the median Foxp3 density. OS, overall survival; HR, hazard ratio; CI, confidence interval; NMI, nonmuscle invasive bladder cancer; Min muscle invasive bladder cancer; TURBT, transurethral resection of bladder tumor; RC, radical resection of bladder cancer.
