# Supplementary figures and images for: Sphingosine 1 phosphate receptor-1 (S1P1) promotes tumor-associated regulatory T cell expansion: leading to poor survival in bladder cancer
Source: Cell Death Dis. 2019 Jan 18;10(2):50. doi: 10.1038/s41419-018-1298-y (PMC6362099; doi:10.1038/s41419-018-1298-y)

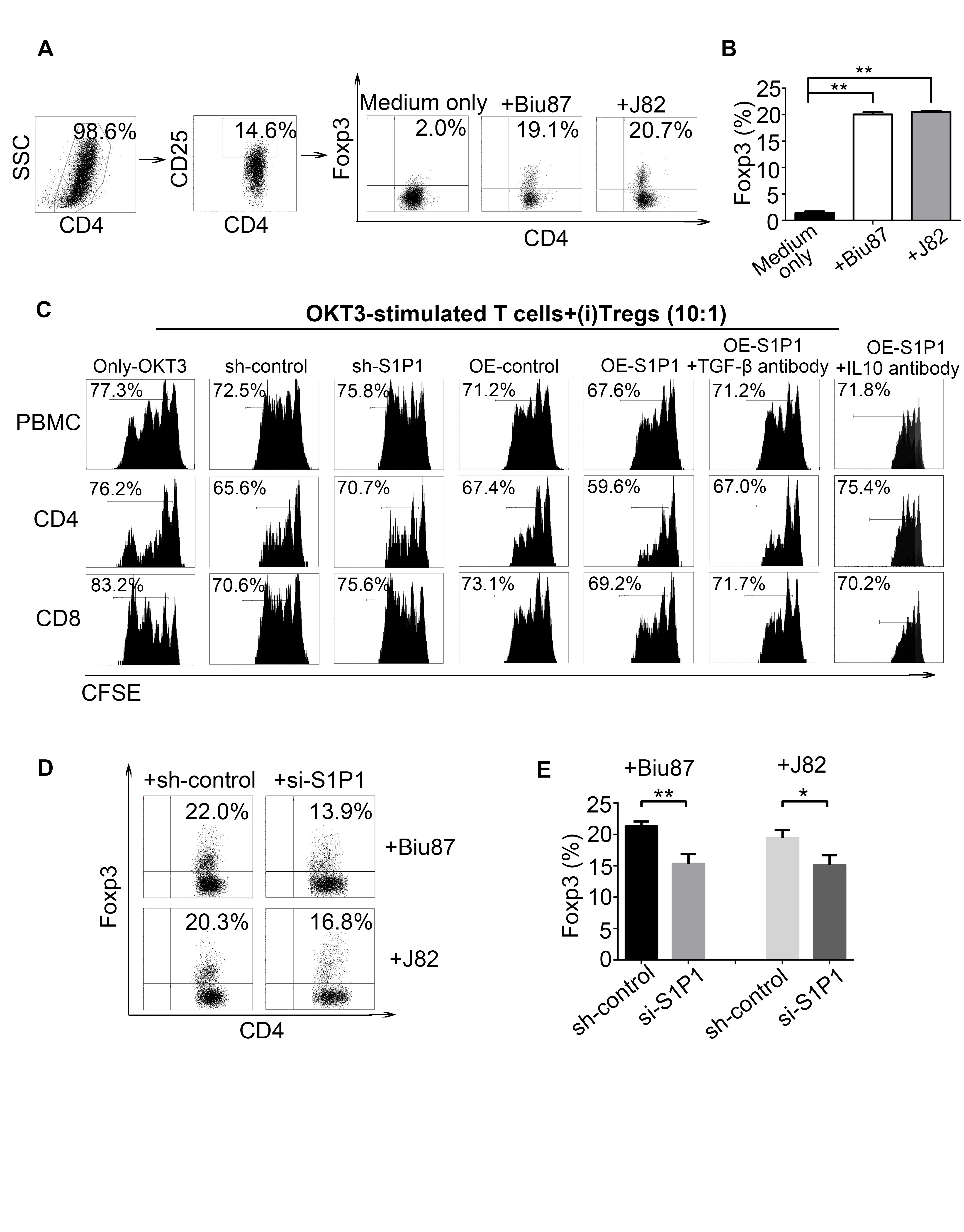

Supplement: Supplementary file 2 — Figure S1 Generation and function of tumor-associated (i)Tregs in BC [file 41419_2018_1298_MOESM2_ESM.jpg]

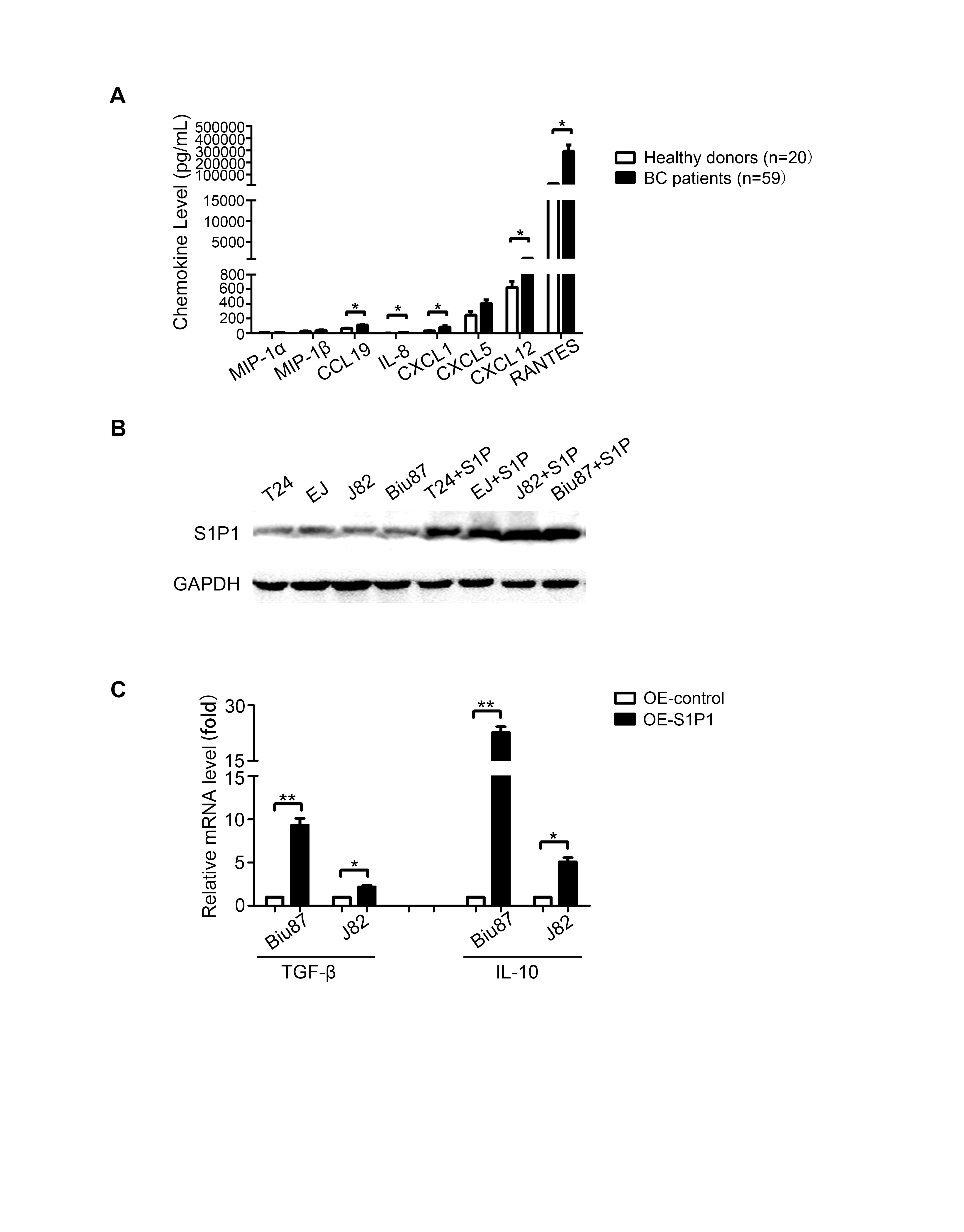

Supplement: Supplementary file 3 — Figure S2 Factors contributing to Treg recruitment in BC [file 41419_2018_1298_MOESM3_ESM.jpg]

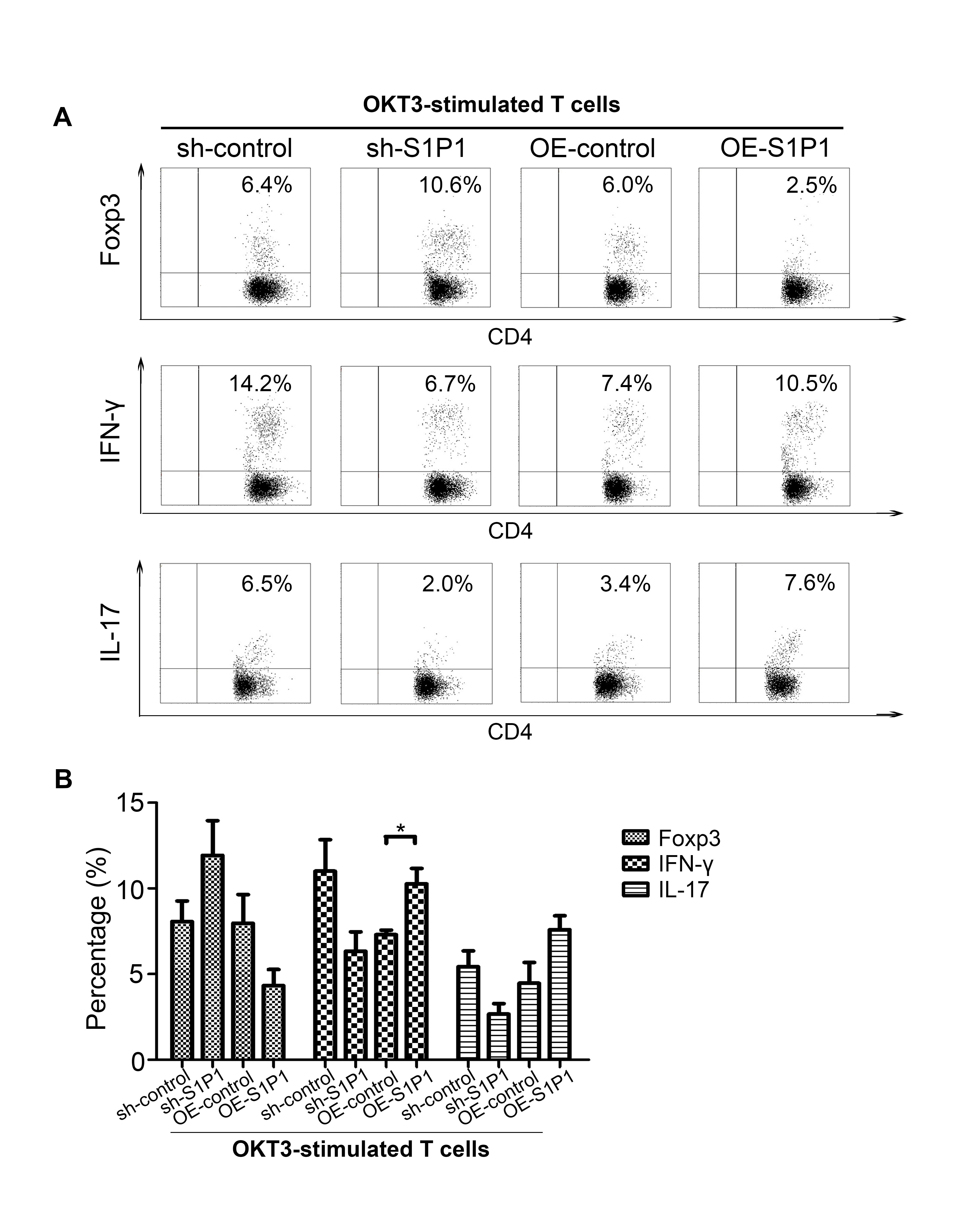

Supplement: Supplementary file 4 — Figure S3 Forced expression or depletion of S1P1 in T cells alters the differentiation of OKT3-stimulated T cells into Th1, Th17 and Treg cells [file 41419_2018_1298_MOESM4_ESM.jpg]
